# Modulation of intracellular kinase signaling to improve TIL stemness and function for adoptive cell therapy

**DOI:** 10.1002/cam4.5095

**Published:** 2022-08-26

**Authors:** Hao Feng, Ling Qiu, Zixiao Shi, Yao Sheng, Peipei Zhao, Di Zhou, Fei Li, Hailin Yu, Yanan You, Hui Wang, Ming Li, Shurong Zhu, Yan Du, Jun Cui, Jingwei Sun, Yarong Liu, Hua Jiang, Xin Wu

**Affiliations:** ^1^ Department of Gynecology Obstetrics and Gynecology Hospital of Fudan University Shanghai People's Republic of China; ^2^ Grit Biotechnology Co., Ltd. Shanghai Shanghai People's Republic of China

**Keywords:** AKT inhibitor, AKT KO, PI3K inhibitor, TIL manufacture, tumor‐infiltrating lymphocyte therapy

## Abstract

**Introduction:**

Adoptive cellular therapy with tumor‐infiltrating lymphocytes (TIL) has demonstrated promising clinical benefits in several solid tumors, but the efficacy of this therapy might be compromised by the “prone‐to‐exhaustion” phenotype of TIL and poor persistence in vivo. This calls for a robust expansion process to produce a large number of cells for clinical usage while at the same time maintaining favorable anti‐tumor function and memory phenotype. Previous studies showed that the PI3K‐AKT signaling pathway plays a key role in the regulation of T cell activation, differentiation and memory formation.

**Method:**

We modulated the PI3K‐AKT pathway in TIL isolated from cervical and ovarian cancer by application of AKT or PI3K inhibitors or CRISPR knockout of AKT1 and/or AKT2, and characterized their effects on TIL phenotype and effector function. Mechanistic study was further performed with RNA‐seq analysis of AKT1/2 KO TIL in comparison to control TIL.

**Result:**

The inhibition of either PI3K or AKT led to an increase in the population of effector CD8^+^ T cells with upregulation of activation markers, elevated CD39^−^CD69^−^ memory T cells, and significantly enhanced cytotoxicity when cocultured with tumor cell lines and patient‐derived tumor samples. Moreover, dual knockout of AKT1 and AKT2 largely phenocopies the functional impact of AKT or PI3K inhibition on TIL. This result was further validated by RNA‐seq analysis indicating that AKT1/2 ablation primarily regulates T cell differentiation and function‐related programs.

**Conclusion:**

Modulation of PI3K‐AKT signaling represents a promising strategy to enhance TIL stemness and cytotoxicity and improve the clinical outcome of current TIL‐based therapy to treat solid tumors.

## Graphical abstract

The PI3K‐AKT pathway in TIL was modulated by AKT or PI3K inhibitors or CRISPR knockout of AKT1 and/or AKT2. The inhibition of either PI3K or AKT led to an increase in the population of effector CD8+ T cells with upregulation of activation marker, elevated CD39CD69 memory T cells, and significantly enhanced cytotoxicity when cocultured with tumor cell lines and patient‐derived tumor samples. The results indicating that modulation of PI3K‐AKT signaling represents a promising strategy to enhance TIL stemness and cytotoxicity and improve the clinical outcome of current TIL‐based therapy to treat solid tumors.

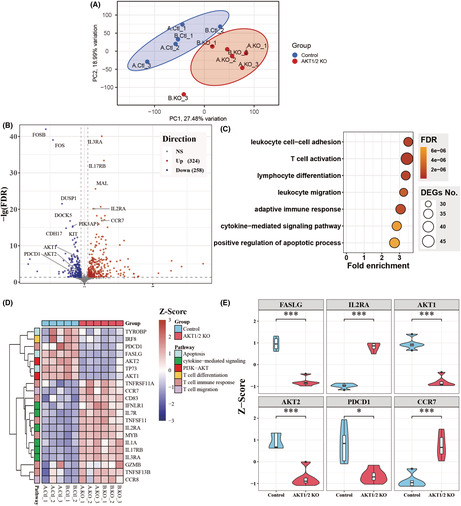

## 1 INTRODUCTION

Adoptive cellular therapy with tumor‐infiltrating lymphocytes has emerged as one of the most powerful therapies for late‐stage solid tumors. TIL was collected from tumors of a patient through biopsy or surgery, amplified in culture with interleukin‐2 (IL‐2) to a clinically relevant level and then reinfused back intravenously to the patient preconditioned with a lymphodepleting regimen. Compared with CAR‐T cell therapy showing remarkable clinical efficacy for certain hematological malignancies, TIL therapy holds unique advantages for treating solid tumors, including its diverse TCR clonality to recognize heterogeneous tumor antigens, superior homing capacity to the tumor site and low off‐target toxicity.^1^ In line with this, durable clinical response rates of 40%–50% have been reported in metastatic melanoma.^2^ Recently, a phase 2 trial of TIL therapy showed an objective response rate (ORR) of 44.4% in cervical cancer patients treated with multiple lines of conventional therapies.^3^ Moreover, TIL therapy demonstrated promising efficacy in patients with progressive disease following anti‐PD‐1 therapy: 36.4% ORR in melanoma patients^4^ and more than 20% in patients with metastatic non‐small cell lung cancer (NSCLC).^5^, ^6^

The attributes of TIL are a major factor contributing to the clinical outcomes after adoptive cell transfer. In vitro cytotoxicity of TIL against autologous or HLA‐matched tumor cells has previously been associated with clinical response.^7^ On the other hand, longer telomeres and higher persistence in peripheral blood 1 month after transfer is correlated with better objective responses,^8^ indicating that a memory‐like phenotype might contribute to clinical efficacy. However, TIL is intrinsically prone to an exhaustion phenotype after being repeatedly stimulated by tumor antigens in the tumor site. Moreover, current manufacturing methods focus primarily on expanding TIL to large numbers, especially for tumor samples with limited T cell infiltration as starting material. This procedure could result in driving TIL to a more differentiated or exhausted phenotype. Indeed, the high dose IL‐2 commonly used in the culture for TIL expansion was shown to promote TIL exhaustion.^9^ Therefore, modification of the current TIL manufacturing process in a way that would uncouple TIL expansion and differentiation would be highly desirable.

The PI3K/AKT pathway is a major signaling pathway mediating T cell proliferation and differentiation in response to T cell activation signals, including TCR, costimulation and cytokine signaling.^10^, ^11^ Previous studies showed that inhibition of AKT, or genetic deletion of the p110δ subunit of PI3K, had no effect on T cell expansion or survival.^12^ A less differentiated T cell phenotype and enhanced anti‐tumor efficacy in a mouse model was observed in CAR‐T cells after pharmacologic inhibition of AKT signaling without compromising T cell expansion.^13^ Similarly, AKT inhibition was shown to expand TIL from melanoma patients with a memory phenotype and improved persistence after adoptive transfer in vivo.^14^ Nevertheless, previous studies of AKT inhibition on cellular therapy mainly focus on memory and metabolic profile characterization. Therefore, the phenotypic and functional profile of TIL after modulation of the PI3K/AKT pathway, especially its anti‐tumor cytotoxicity, remains to be fully explored to support its potential application in large‐scale TIL manufacturing process. Furthermore, it is not known if a similar effect would be seen in TIL from solid tumors apart from melanoma. Here, we studied the functional impact of modulating the PI3K/AKT pathway in TIL from cervical and ovarian cancer by applying PI3K and AKT inhibitors or dual KO of AKT1/2. Mechanistic study was subsequently performed by RNA‐seq analysis of AKT1/2 KO TIL compared to control TIL.

## 2 RESULTS

### 2.1 Pharmacologic inhibition of PI3K or AKT signaling promotes TIL proliferation and stemness

**FIGURE 1:**
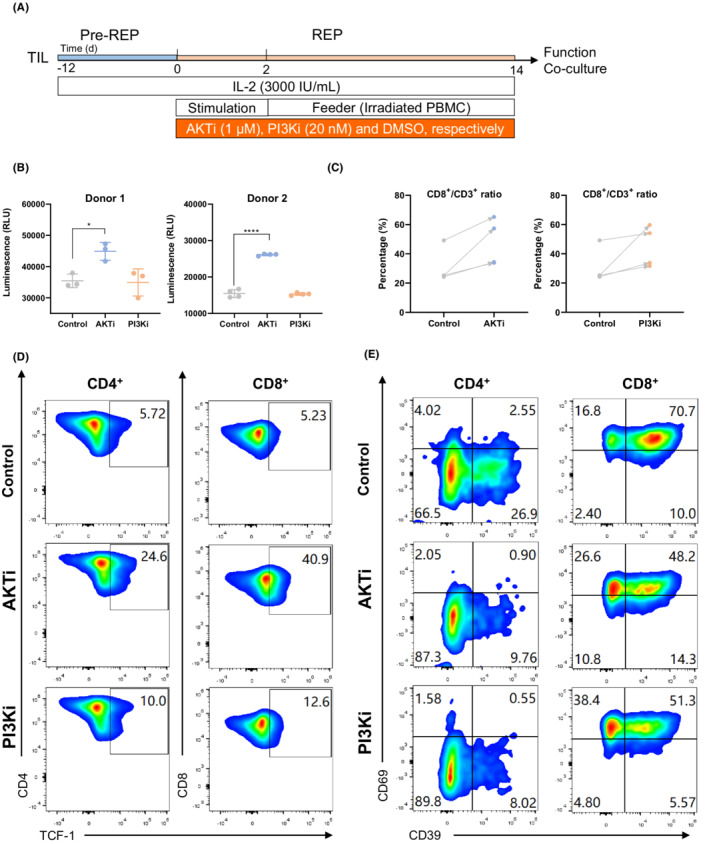
AKT or PI3K inhibitors promote TIL proliferation and stemness. (A) A schematic representation of Pre‐REP and REP of human TIL in the continuous presence of IL‐2 (3000 IU/ml), 1 μM AKTi or 20 nM PI3Ki; (B) After REP expansion, TIL was re‐plated in a 96‐well plate with the same concentration of AKTi or PI3Ki, but no IL‐2, for 72 h, and proliferation of TIL was analyzed by CellTiter‐Glo assay (CTG). Data are mean ± s.d.. *P‐*values by two‐tailed unpaired *t*‐test *P < 0.05, ****P < 0.0001; (C) Ratio of CD8+ and CD4+ in CD3+ TIL at the end of REP; (D and E) Expression of TCF1, CD39 and CD69 on CD8+ or CD4+ TIL, as analyzed by flow cytometry.

TIL was expanded from cervical and ovarian tumors with the manufacturing procedure as shown in Figure 1A. In brief, tumor surgical specimens were minced into fragments, and TIL was cultured in a previous rapid expansion phase (preREP) with high dose IL‐2, followed by amplification to a large number in a rapid expansion phase (REP).

We first sought to confirm whether PI3K or AKT inhibition would impact the expansion and memory phenotype of TIL. REP of TIL was performed in the continuous presence of 1 μM of AKT inhibitor (AKT Inhibitor VIII; AKTi) or 20 nM PI3K inhibitor (idelalisib; PI3Ki) with 3000 IU/ml IL‐2. No significant influence of PI3K or AKT inhibition was observed on TIL expansion during REP in three independent donors (Figure S1B). However, application of AKTi, but not PI3Ki, significantly promoted the proliferation of the final TIL product post REP stage where IL‐2 had been withdrawn (Figure 1B). This result suggests that the proliferation advantage endowed by AKTi might be overridden by high‐dose IL‐2 during REP stage. Moreover, both AKTi and PI3Ki could promote enrichment of the CD8^+^ T cell population (Figure 1C), but not the CD4^+^ T cell population (Figures S1C) in four different donors.

The transcription factor TCF1, a marker for stem cell‐like T cells, plays a critical role in inducing and maintaining T cell stemness as well as preserving CD8^+^ T cell functionality upon exposure to tumor antigens or viral infection.^15^, ^16^ Either AKTi or PI3Ki could increase TCF‐1 expression in both CD4^+^ and CD8^+^ T cell populations (Figure 1D; Figure S1D). In addition, a memory‐progenitor stem‐like phenotype (CD39^−^CD69^−^) plays a critical role in TIL persistence and complete cancer regression in patients.^17^ Tumor‐reactive CD39 − CD69− TILs were capable of self‐renewal and expansion and demonstrated superior antitumor response and persistence in vivo. This TIL subset was increased in both CD4^+^ and CD8^+^ T cell populations after PI3K or AKT inhibition compared to the control group (Figure 1E; Figure S1E), indicating its higher stemness and potential better *in vivo* efficacy and persistence. Therefore, PI3K/AKT pathway inhibition promotes TIL proliferation and stemness and facilitates the enrichment of CD8^+^ T cells.

### 2.2 AKT or PI3K inhibitor increases TIL activation and cytotoxicity

**FIGURE 2:**
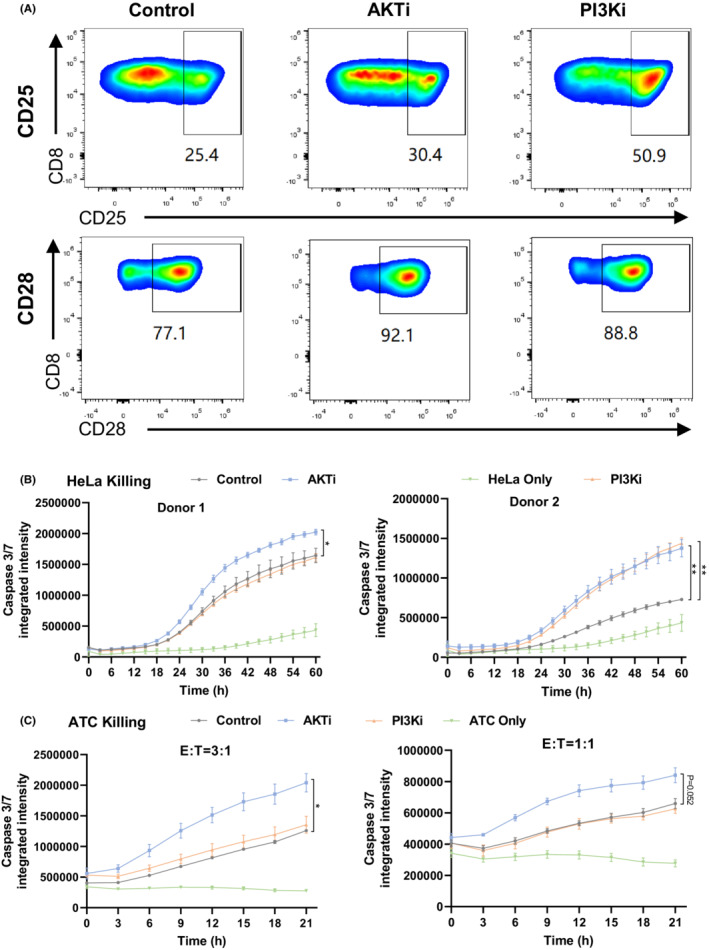
AKT or PI3K inhibition enhances TIL activation and cytotoxicity. (A) The expression of CD25 and CD28 on CD8+ TIL population analyzed by flow cytometry; (B) AKTi or PI3Ki‐treated TIL from two cervical cancer patients was co‐cultured with HeLa cells at an effector to target ratio (E:T) 1:1 and real‐time tumor killing was indicated with Caspase 3/7 signal monitored by IncuCyte; (C) The tumor killing curve of AKTi or PI3Ki‐treated TIL from a cervical cancer patient against autologous tumor cells at E:T = 3:1 and E:T = 1:1. Data are mean ± s.e.m.. *P‐*values by two‐way ANOVA. *P < 0.05, **P < 0.01.

Next, we further examined the effect of PI3K and AKT inhibitors on TIL functionality. TIL with exposure of PI3K and AKT inhibitors showed increased CD25 and CD28 expression in the CD8^+^ T cell population (Figure 2A), suggesting a stronger TIL activation status upon inhibition. Exhaustion markers, including PD‐1, LAG‐3, TIM‐3, CD38 and CD101, were also analyzed on the final TIL product and no significant difference was observed between the treated or non‐treated group (data not shown). The tumor‐killing ability of TIL was performed on both HeLa cell line and autologous tumor cells (ATC) from a cervical cancer patient. TIL with AKT inhibition showed significantly stronger cytotoxicity against both HeLa and ATC compared with the control TIL group. In contrast, PI3K inhibition only showed improved TIL cytotoxicity to HeLa cells in one donor, but no effect on TIL killing against ATC (Figure 2B,C), indicating AKTi may play a more important role in the regulation of TIL function compared with that of PI3Ki.

### 2.3 AKT1/2 double KO enhances TIL proliferation and stemness

**FIGURE 3:**
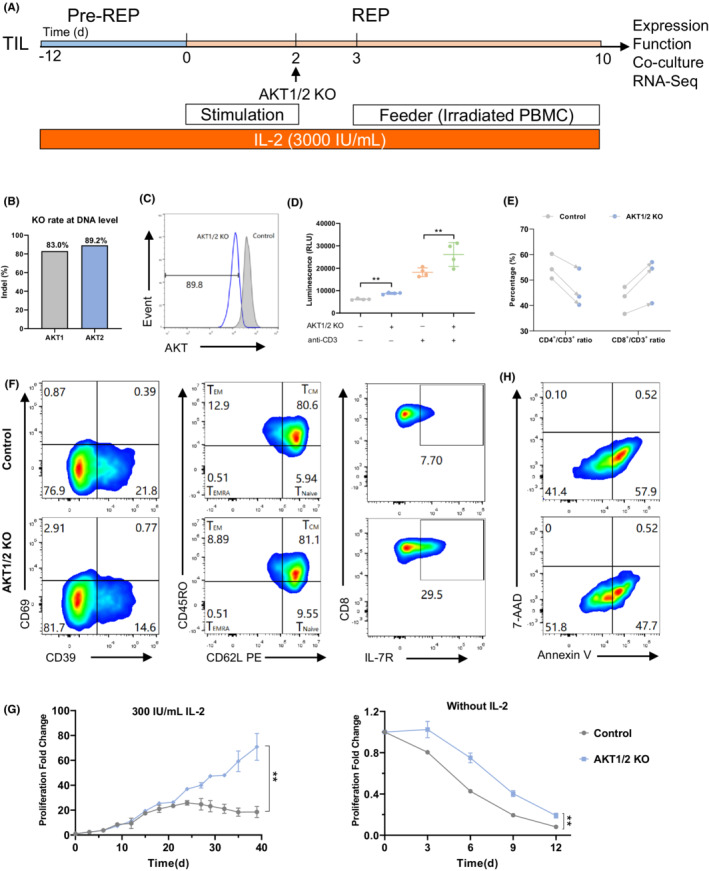
AKT1 and AKT2 double KO facilitates TIL proliferation and stemness. (A) Schema for the Pre‐REP, AKT1/2 KO and REP of human TIL in the continuous presence of IL‐2 (3000 IU/ml); (B) KO efficiency of AKT1 or AKT2 analyzed using TIDE based on sanger sequencing; (C) AKT1/2 protein expression in AKT1/2 KO TIL versus control TIL by flow cytometry; (D) After REP expansion, AKT1/2 KO or mock transfected TIL was plated in a 96‐well plate with or without a‐CD3 antibody stimulation as indicated for 72 h and proliferation of TIL was analyzed by CTG. Data are mean ± s.d.. *P* values by two‐tailed unpaired *t*‐test. **P < 0.01; (E) The ratio of CD8+ and CD4+ in CD3+ TIL at the end of REP from two treatment groups; (F) The expression of indicated markers on CD8+ TIL analyzed by flow cytometry. T_EM_, effect memory T cell; T_CM_, central memory T cell; T_Naive_, naive T cell; T_EMRA_, effector memory cells with expression of CD45RA; (G) Proliferation of AKT1/2 KO or control TIL in culture in the presence of 300 IU/ml IL‐2 or no IL‐2 after REP stage. Data are mean ± s.d. *P‐*values by two‐way ANOVA. **P < 0.01; (H) Apoptosis analysis of CD8^+^ TIL by flow staining of 7‐AAD and Annexin V.

Given the stronger functional impact of AKTi compared to that of PI3Ki, we further studied whether AKT knockout could lead to a similar functional benefit in TIL. Through a screening experiment, guide RNAs (gRNA) with the highest knockout efficiency were selected from 8 gRNA candidates (data not shown). AKT1 or AKT2 was knocked out independently using CRISPR/Cas9 technology with mock transfected TIL as a control (Figure 3A). Both AKT1 and AKT2 single KO upregulated the expression of IL‐7R on CD4^+^ and CD8^+^ TIL, but no effects were observed on TIL expansion, stemness, activation or cytotoxicity (Figure S2).

As AKT Inhibitor VIII is a potent selective inhibitor of AKT1 and AKT2 with IC50 values of 58 nM and 210 nM, respectively,^18^ we hypothesized that AKT1 or AKT2 KO may not have sufficiently blocked AKT signaling in the previous experiment. Therefore, we generated AKT1 and AKT2 double knockout (AKT1/2 KO) TIL from cervical cancer and ovarian cancer using the procedure shown in Figure 3A. Genome editing efficiency of AKT1 and AKT2 genes was 80.3% and 89.2%, respectively (Figure 3B). Total AKT expression in TIL was reduced by 89.8% after AKT1/2 KO, as indicated by flow cytometry (Figure 3C).

To test the effect of AKT1/2 KO during REP stage, the final product of AKT1/2 KO, or control TIL was cultured with or without anti‐CD3 restimulation. AKT1/2 KO significantly promoted TIL proliferation either with or without anti‐CD3 compared to the control group (Figure 3D). Like AKTi, AKT1/2 KO facilitated CD8^+^ T cell expansion over that of the CD4^+^ T cell population during REP stage in TIL from 3 different donors (Figure 3E). Moreover, AKT1/2 KO increased the proportion of CD39^−^CD69^−^ memory T cell and naive T cell (CD45RO^−^ CD62L^+^) population as well as IL‐7R expression in TIL (Figure 3F; Figure S3). To further determine the influence of AKT1/2 KO on TIL memory and persistence, we performed a long‐term culture of TIL after REP stage with no, or low, dose of IL‐2. Results showed that AKT1/2 KO significantly increased the long‐term survival of TIL without or with 300 IU/ml IL‐2 (Figure 3G). In addition, AKT1/2 KO reduced early apoptotic in CD8^+^ TIL (Figure 3H). Therefore, AKT1/2 KO showed effects consistent with those of AKT inhibition on CD8 T cell enrichment and memory phenotype preservation, as well as enhanced TIL long‐term persistence in vitro.

### 2.4 AKT1/2 KO enhances TIL activation, cytokine production and cytotoxicity

**FIGURE 4:**
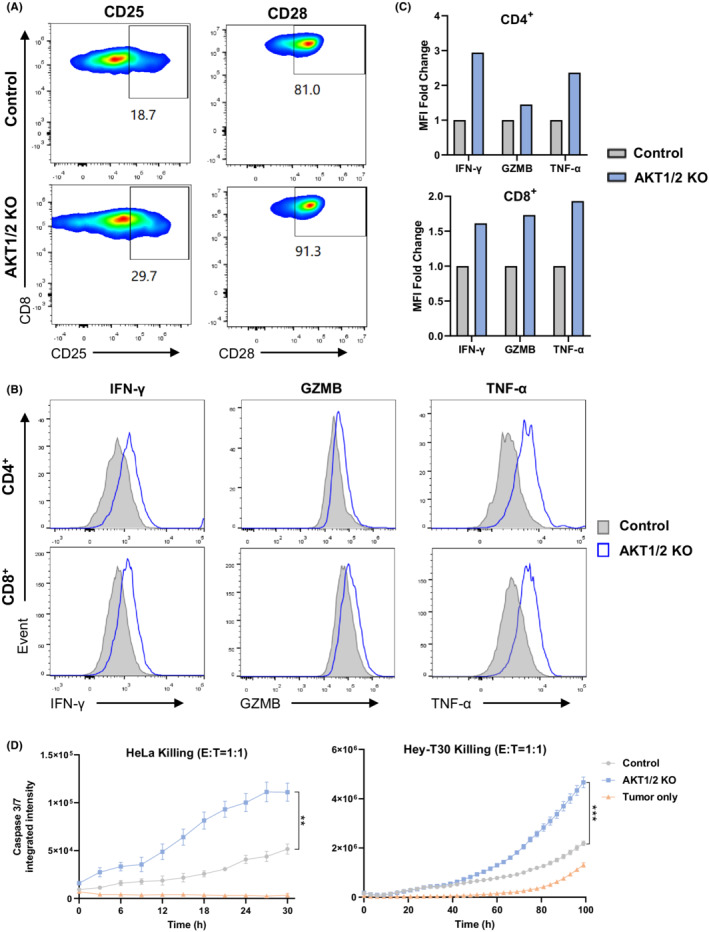
AKT1/2 KO TIL demonstrated enhanced activation and effector function. (A) The expression of CD25 and CD28 on CD8+ TIL by flow staining; (B, C) IFN‐γ, GZMB and TNF‐α production in TIL from a cervical cancer patient was analyzed by flow cytometry after a‐CD3 restimulation overnight; (D) The tumor killing curve of AKT KO and control TIL against HeLa or Hey‐T30 cell lines at E:T = 1:1. Data are mean ± s.e.m.. *P*‐values by two‐way ANOVA. **P < 0.01, ***P < 0.001.

We further tested whether AKT1/2 KO could promote anti‐tumor function of TIL in a manner similar to that seen in AKT inhibition. AKT1/2 KO increased CD25 and CD28 expression in both CD4^+^ and CD8^+^ TIL (Figure 4A; Figure S4A), which is consistent with the effect of AKTi. Further analysis showed that AKT1/2 KO promoted IFN‐γ, GZMB and TNF‐α production in both CD4^+^ and CD8^+^ T cell subsets in response to anti‐CD3 restimulation (Figure 4B,C; Figure S4B,C). Moreover, AKT1/2 KO enhanced tumor killing of TIL against tumor cell lines HeLa or Hey‐T30 compared with the control group (Figure 4D).

### 2.5 AKT1/2 KO facilitates the transcriptional program associated with T cell function and memory in TIL

**FIGURE 5:**
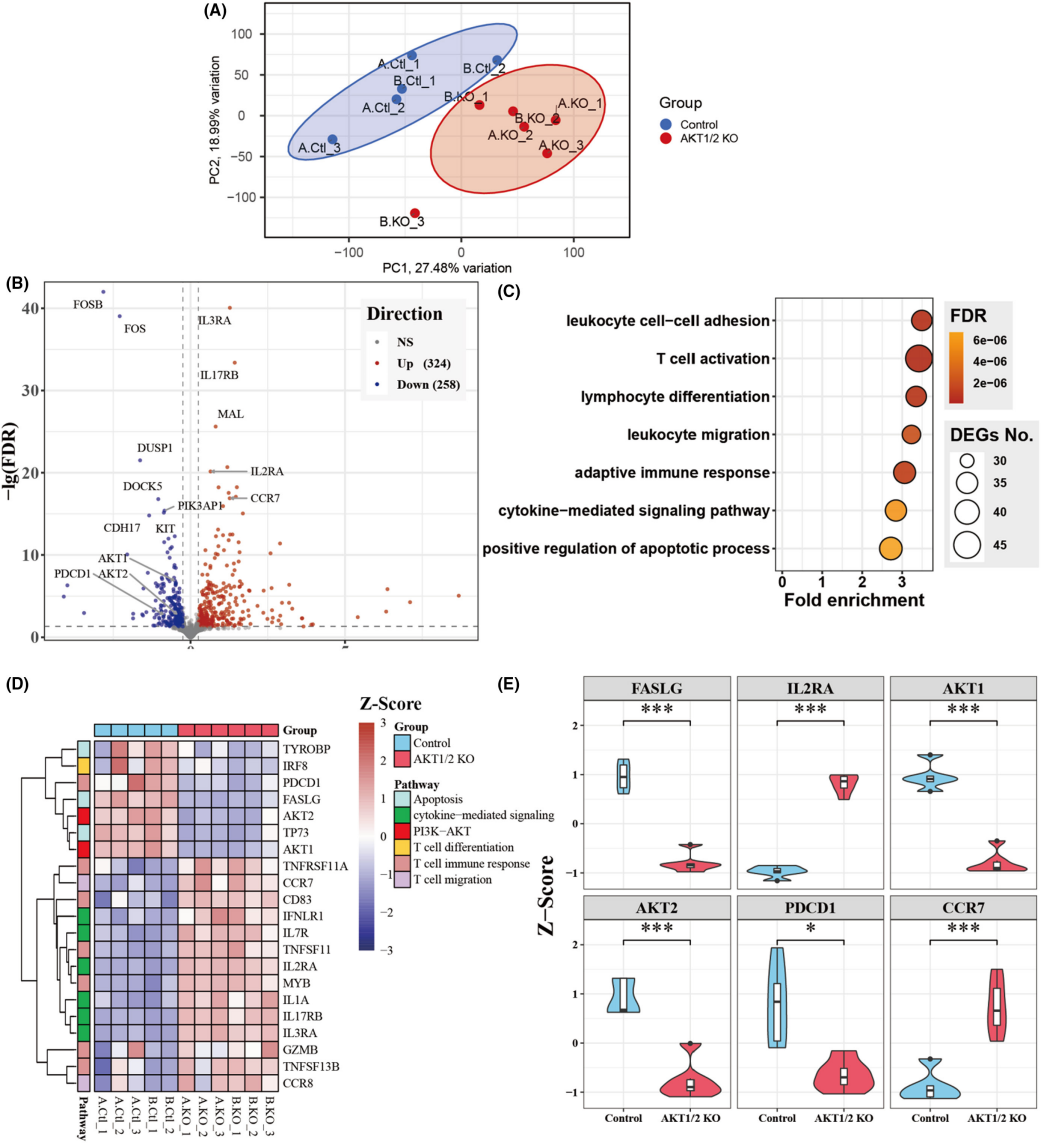
AKT1/2 KO facilitates transcriptional program of T cell function and memory in TIL. (A) PCA plot of DESeq2 adjusted gene expression data. Ellipses with 95% confidence by the group were shown. (B) Volcano plot of up‐ or down‐regulated DEGs in AKT1/2 KO group compared to control group. X‐axis indicates the log2 Fold Change of gene expression in KO versus control TIL. Y‐axis indicates the ‐log10 adjust *P*‐value (FDR). (C) Go enrichment dot plot of significantly different biological processes between control and KO TIL. Fold enrichment is obtained through the number of DEGs found in a specific process divided by the number of DEGs expected to be found. The colors of the dots represent the adjusted *P*‐value (FDR) of the processes. The size of the dots indicates the number of matched DEGs in the process. (D) Z‐score heatmap of representative DEGs involved in PI3K − AKT related biological processes. Red and blue colors indicate higher and lower scaled expression levels. (E) Violin and boxplot showing the relative expression of 6 DEGs. The *P*‐value of Z scores was calculated by Wilcoxon's Rank Sum test, two‐sided. The significant level of *P*‐value was shown. *P < 0.05, **P < 0.01, ***P < 0.001.

Having shown that pharmacologic inhibition or CRISPR KO of AKT in TIL promotes T cell memory and effector function, we further explored the transcriptional programs regulated by AKT in human TIL. The transcriptome of AKT1/2 KO and mock transfected TIL was analyzed by RNA‐seq. To visualize the transcriptome between the two groups, we performed principal component analysis (PCA) of the RNA‐seq data. It showed clear segregation between AKT1/2 KO and control groups between the 2 donors (Figure 5A). Whole‐transcriptome analysis revealed 582 differentially expressed genes (DEGs) (Table S1) out of 23,209 detected genes, and the DEGs are illustrated in volcano plot with |log2FC| > 0.25 and FDR <0.05 as cutoff (Figure 5B). AKT1 and AKT2 were significantly downregulated in the AKT1/2 KO group, validating the efficiency of CRISPR KO in TIL. To understand the biological pathways in which the DEGs are involved in, we applied analysis of Gene Ontology Biological Processes (GO BP) and observed enrichment of these genes in lymphocyte activation, differentiation, and apoptosis, as well as cytokine‐related signaling (Figure 5C). Unsupervised clustering of the representative genes in these pathways demonstrated segregation between AKT1/2 KO and control TIL (Figure 5D). We observed significantly upregulated CCR7 and IL2RA and downregulated PDCD1 and FASLG was observed in AKT1/2 KO TIL, which is consistent with our findings that AKT1/2 KO enhances TIL memory and function, but prevents apoptosis (Figure 5E).

T cell metabolism, such as glycolysis and mitochondrial fatty acid oxidation has been associated with T cell memory and effector function.^19^, ^20^ Thus, we investigated the transcriptional profile of related genes in these two pathways.^14^, ^21^, ^22^, ^23^, ^24^ In line with the previous study on AKTi‐treated melanoma TIL, altered expression of lipid metabolism related‐genes was observed in AKT1/2 KO TIL, but no difference was seen in glycolysis pathway (Figure S5A,B). In contrast to the PI3K/AKT pathway that was significantly downregulated after AKT1/2 KO (FDR = 8.093e‐05), the MAPK pathway, another key pathway mediating T cell activation/function signaling, showed no significant alteration between the two groups (FDR = 0.061, Table S2). This result suggests that PI3K/AKT pathway disruption may not impact MAPK pathway shown to be essential for T cell proliferation and survival, a result that is also consistent with previous study of AKTi in CAR‐T cells.^13^

## 3 DISCUSSION

Considering the promising efficacy of TIL in metastatic solid tumors, refining the current manufacturing methodologies to improve TIL function and stemness without sacrificing of its expansion potential may benefit clinical outcomes of TIL therapy in multiple late‐stage solid tumors. In this study, we have shown that application of AKT or PI3K inhibitors during REP stage of TIL from cervical or ovarian cancer favors generation of the effector CD8+ T cell population with higher activation marker expression and more TCF‐1+ and CD39‐CD69‐ memory T cells in both CD4+ and CD8+ T cell populations, even while failing to impact, or even enhance TIL proliferation post‐REP. On the other hand, AKTi or PI3Ki did significantly increase TIL cytotoxicity in the coculture with tumor cell lines and autologous tumor cells. Furthermore, dual knockout of AKT1/2 in TIL during REP largely phenocopies AKT inhibition, as further confirmed by the RNA‐seq analysis. These findings have positive implications in overcoming the current limitations of TIL therapy in solid tumors.

A high proportion of CD8+ T cells in the final TIL product,^25^ in vitro cytotoxicity against autologous tumor cells and in vivo persistence of TIL post transfer have been associated with improved clinical response in metastatic melanoma. AKT inhibition or deletion does increases (1) the percentage of CD8+ T cells at the end of REP, (2) cytokine production including IFN‐γ, TNF‐α and GZMB production upon restimulation in response to a‐CD3/a‐CD28, and (3) tumor killing ability of TIL to patient‐derived tumor cells, all promising better anti‐tumor efficacy of TIL in patients. Moreover, enhanced T cell memory phenotype and long‐term survival with no, or low, dose of IL‐2 and decreased apoptosis after AKT inhibition or ablation may lead to better persistence of TIL in vivo. Taken together, modulation of AKT during TIL's rapid expansion stage might contribute to a better clinical outcome, which would be especially meaningful for ovarian or cervical cancer that generally have a lower response rate of TIL‐based therapy compared to melanoma.

In the present study, tumor reactivity of TIL after AKT inhibition or KO was significantly increased compared to unmodified TIL in in vitro co‐culture systems. In vivo study will further validate our findings of AKT ablation on anti‐tumor function and memory formation of TIL. The in vivo function of these TIL could be further examined with a patient‐derived xenograft model (PDX) to support its anti‐tumor efficacy, which is under development. The diversity of the TCR repertoire may be a predictive marker of clinical response,^26^, ^27^ which reflects the potential of T cells to recognize heterogenous tumor antigens. Some TCR clones might be lost during rapid expansion in response to allogenic PBMC feeders and high‐dose IL‐2. Accordingly, it would be interesting to test whether modulation of AKT pathway could better preserve TCR clonality of TIL during REP stage.

The PI3K/AKT/mTOR signaling pathway plays a critical role in cell growth, survival and glucose metabolism. Recent studies also highlighted the importance of this pathway in the regulation of CD8+ T cell memory formation and differentiation.^10^, ^11^, ^12^ In our study, AKT inhibition leads to more dramatic functional impact on TIL compared to PI3Ki in terms of CD8+ T cell population enrichment, TCF1 upregulation and cytotoxicity against autologous tumor cells or HeLa cells. This is consistent with previous findings of AKT as an essential coordinator of gene transcription upon T cell activation to distinguish memory and effector T cell formation.^28^ Moreover, single KO of AKT1 or AKT2 caused no significant functional improvement of TIL, indicating that the compensation occurs between these two AKT isoforms despite their nonoverlapping functions. It's noted that previous studies show AKTi could decrease the production of effector molecules, such as perforin and IFN‐γ, but rather sustain a memory‐naive phenotype,^12^, ^13^ while here we found ATK1/2 ablation promoted TIL activation and effector function as revealed by increased CD25 and CD28 expression as well as IFN‐γ and GZMB production. The difference might be explained by the different T cell types—the human PBMC or mouse splenic T cells used in previous studies are at different stages from TIL derived from patient tumors and undergoing distinct expansion process. Moreover, different AKT modulation strategies, AKT KO verse AKT inhibitors, may also behave differently in terms of the completeness and specificity of AKT inhibition. In addition, we have found that inhibition of mTOR downstream of AKT resulted in growth arrest of TIL, which is consistent with the key role of mTOR in T cell survival and proliferation.^29^ Taken together, these findings validated the importance of AKT as a crucial regulator of proliferation versus memory aspect of antigen‐experienced T cells.

In conclusion, we show that pharmacological inhibition or genetic ablation of PI3K/AKT during REP stage of TIL in cervical and ovarian cancer promotes the propagation of tumor‐reactive TIL with memory‐like phenotype. These findings warrant clinical testing of PI3K/AKT modulation to improve the clinical efficacy of TIL therapy for solid tumor patients.

## 4 METHODS

### 4.1 TIL preparation

Tumor surgical specimens were obtained from cervical and ovarian cancer patients between 18 and 70 years old who have at least one tumor lesion with a diameter of more than 1.5 cm, and could be treated with reasonable tumor reduction surgery in accordance with NCCN clinical guidelines. Patients who received radiotherapy within 28 days before surgery were excluded. This study was approved by the Medical Ethics Committee of Obstetrics and Gynecology Hospital of Fudan University, with the Approval number 2021–07.

Primary tumors or lymph node metastases were minced into fragments (1–20 mm^3^) and placed into a Grex‐100 for PREREP culture. At the stage of REP, TIL was initially stimulated with 30 ng/ml of ANTI‐CD3 antibody (OKT3; ORTHOBIOTECH) for 48 h, followed by addition of feeders (irradiated PBMC, 200 folds of TIL). At both stages, TIL was maintained in T cell media with 3000 IU/ml RHIL‐2 (T&L Biotechnology).

### 4.2 Cell culture

Tumor cell lines (HeLa and Hey‐T30, ATCC) and patient derived tumor cells were maintained in D10 medium, which is consisted of DMEM medium (Gibco) supplemented with 10% FBS (Gibco), and 1% penicillin/streptomycin (Gibco). TIL was maintained in T cell media with 3000 IU/ml IL‐2 during Pre‐REP and REP stage. At the stage of REP, TIL was initially stimulated with 30 ng/ml of anti‐CD3 antibody (OKT3, OrthoBiotech) for 48 h, followed by adding feeder cells (irradiated PBMC, 200 folds of TIL). PI3Ki‐ or AKTi‐ treated TIL was cultured continuously with 1 μM of AKT Inhibitor VIII (AKTi) (MedChemExpress, HY‐10355) or 20 nM PI3Ki (idelalisib) (MedChemExpress, HY‐13026) dissolved in 0.1% DMSO (MilliporeSigma) in accordance with a previous study.^13^

### 4.3 Cas9/RNP nucleofection

Deletion of AKT1 and/or AKT2 in TIL was performed using CRISPR/Cas9 technology. Precomplexing of Cas9/RNP was done in accordance with previous methods before nucleofection.^30^ Briefly, 20 μM sgRNA and 4 μM cas9 protein diluted in primary cell nucleofection solution P3 (Primary Cell 4D‐Nucleofector X kit S [32 RCT, V4XP‐3032; Lonza]) were incubated at 25°C for 10 min to form Cas9/RNP. TIL of Pre‐REP was stimulated with TransAct (Miltenyi Biotec) for 48 h. One million TIL was resuspended in 20 μl primary cell nucleofection solution P3 and mixed with 5 μl Cas9/RNP. Cells were electroporated by a 4D nucleofector (4D‐Nucleofector Core Unit, Lonza). After nucleofection, feeder cells (irradiated PBMC, 200 folds of TIL) were added for TIL expansion. By KO efficiency screening of 8 guides, the top sgRNAs with highest AKT1 or AKT2 KO efficiency were selected for later experiments (AKT1: GACAACCGCCATCCAGACTG, AKT2: GACCCCATGGACTACAAGTG). Genome DNA of TIL was extracted with QuickExtract DNA Extraction Solution (QE09050, Lucigen). DNA sequence of editing area was obtained by PCR and sanger sequencing was performed after purification. AKT1/2 KO efficiency at DNA level was analyzed by the method of Tracking of Indels by Decomposition (http://shinyapps.datacurators.nl/tide/).

### 4.4 Antibodies and flow cytometry

Cells were stained with fluorochrome‐conjugated antibodies against combinations of the following antigens: CD3 (740187), CD45 (557748), CD4 (563737), CD8 (564,526 or 562,428), CD107A (562623), CD45RO (555492), CD62L (555544), PD‐1 (563789), TIM‐3 (565558), CD38 (562665), CD25 (564467), CD28 (555730) (all from BD Biosciences); IL‐7RΑ (351320), CD101 (331016) (both from Biolegend); (65–0865‐14, EBIOSCIENCE). Flow cytometric data was acquired using a Cytoflex Flow cytometer (BECKMAN). FACS data was analyzed with FLOWJO Version 10.4.0 software (TREESTAR).

### 4.5 AKT expression

TIL was harvested during REP, washed with PBS and then stained with Fixable Viability Dye, CD3, CD4 and CD8 for 30 min at 4°C. After surface staining, the cells were washed with PBS and then permeabilized with Cytofix/Cytoperm (BD Biosciences, 562,574) for 20 min at 4°C. Next, cells were washed twice with wash buffer and stained with ANTI‐AKT antibody (C67E7, Cell Signaling Technologies) for 30 min at 4°C. After washing, cells were resuspended in PBS and analyzed by Cytoflex Flow cytometer (BECKMAN).

### 4.6 CD107a detection, cytokine intracellular staining and apoptosis analysis

TIL was harvested during REP, washed with PBS and plated in a 96‐well plate with 30 ng/ML of ANTI‐CD3 antibody. TIL culture was incubated with ANTI‐CD107A antibody, 1:1000 Golgi Stop and 1:1000 Golgi Plug solutions (554,724, 555,029, BD Biosciences) overnight. Cells were then stained with surface markers and permeabilized as mentioned above. Next, cells were washed and stained with TNF‐α (557,647, BD Biosciences), IFN‐γ (566,394, BD Biosciences) and GZMB (372,207, Biolegend) for 30 min at 4°C. After washing, cells were resuspended in PBS and analyzed by flow cytometer. TIL apoptosis was analyzed with Annexin V apoptosis kit (BD Biosciences, 559,763). Briefly, cells were stained with surface markers as mentioned above. After surface staining, the cells were washed and incubated with Annexin V and 7‐AAD according to manufacturer instruction.

### 4.7 Tumor killing monitor

Target cells (HELA, HEY‐T30 or autologous tumor cells) were seeded in a 96‐well plate at a density of 2000 cells/well 12 hr followed by addition of SUPERVIEW™ 488 Caspase 3/7 Substrate (S6007L, US Everbright) to each well. TIL was added at indicated E:T ratios, and tumor killing was monitored by INCUCYTE (Sartorius) to acquire images every 3 h. Experiments were performed with 3 independent technical replicates.

### 4.8 Proliferation analysis

TIL was harvested, washed with PBS and seeded in a 96‐well plate at density of 1.5E5 cells/well with or without stimulation by 30 ng/ml of ANTI‐CD3 antibody. After 72 h culture, proliferation of TIL was analyzed by CELLTITER‐GLO® Luminescent Cell Viability Assay (G7570, Promega) according to manufacturer instruction. Luminescent value was measured by a microplate reader (Molecular Devices, SPECTRAMAX i3x).

### 4.9 Total RNA extraction

Total RNA was extracted from the tissues using Trizol (Invitrogen, Carlsbad, CA, USA) and RNAprep pure Cell kit DP430 (TIANGEN, Beijing, China) according to manual instruction. Subsequently, total RNA was qualified and quantified using a Nano Drop and Agilent 2100 bioanalyzer (Thermo Fisher Scientific, MA, USA).

### 4.10 mRNA library construction

Oligo(dT)‐attached magnetic beads were used to purify mRNA. Purified mRNA was fragmented into small pieces, and cDNA was generated by Hieff NGS Ultima Dual‐mode mRNA Library Prep Kit for MGI (Yeasen, Shanghai, China) following manual instruction. The reaction product was purified by magnetic beads, followed by addition of A‐Tailing Mix and RNA Index Adapters. The cDNA fragments with adapters were amplified by PCR, and then purified by Ampure XP Beads. The quality and quantity of library was assessed using the Agilent 2100 bioanalyzer, and then underwent DSN treatment. Next, the library was analyzed to ensure the high quality of the sequencing data by Agilent 2100 bioanalyzer and qPCR. The qualified library was amplified on cBot to generate the cluster on the flowcell, and the flowcell was single‐end sequenced on the Illumina Novaseq 6000 platform (Tsingke‐Beijing, China).

### 4.11 RNA sequencing

The sequencing data was filtered with SOAPnuke (v1.5.2) with three criteria: (1) removing reads containing sequencing adapter^31^; (2) removing reads whose low‐quality base ratio (base quality less than or equal to 5) is more than 20%; (3) removing reads whose unknown base (‘N' base) ratio is more than 5%. Then, clean reads were stored in FASTQ format and mapped to the reference genome using HISAT2^32^ (v2.0.4). Bowtie2 (v2.2.5)^33^ was applied to align the clean reads to the reference coding gene set and the expression level of genes was calculated by StringTie^34^ (v2.1.2).

### 4.12 Differential gene expression analysis

R (v4.1.1) and R packages were used to find and analyze differential expressed genes (DEGs). Statistical analysis of DEGs was conducted with DESeq2^35^ (v1.34.0). Apeglm^36^ algorithm was used for effect size shrinkage, and the adjusted counts were used to calculate the log2FC. A false discovery rate (FDR) <0.05 and |log2FC| > 0.25 were used to identify DEGs between control and AKT1/2 KO group. DEGs were used to analyze over‐represented Gene ontology biological processes (GO BP) and KEGG pathways by clusterProfiler (v4.2.0). PCA plot, volcano plots, dot plots, heatmap, violin plots were constructed using PCAtools(2.6.0), ggplot2 (v3.3.5), pheatmap (v1.0.2), ggsignif (v0.6.3), respectively, and custom scripts in R.

### 4.13 Quantification and statistical analysis

Statistical analyses were performed using Prism 8.4 (Graphpad Software). At least three replicates were performed for each experiment. Figure legends specify the statistical analysis method performed. The error bars in the figures represent the standard error of the mean (s.e.m.) or the standard deviation (s.d.) as indicated in the corresponding legends. *P* values were considered statistically significant if *P <* 0.05.

## AUTHOR CONTRIBUTIONS

X.W., H.J., Y.L., J.S., and J.C. conceptualized and designed the study. H.Y., Y.Y., H.W., M.L., and S.Z. contributed to sample preparation. H.F., L.Q., Z.S., Y.S., P.Z., Y.D., and F.L. carried out the experiments. H.F., L.Q., Z.S., Y.S., P.Z., and D.Z. performed analyses. H.F., L.Q., Z.S., and Y.S. wrote the manuscript. X.W., H.J., Y.L., and J.S. reviewed and edited the manuscript. All authors approved the final manuscript.

## CONFLICT OF INTEREST

Z.S., Y.S., P.Z., D.Z., F.L., J.C., J.S., and Y.L. were employed by the company Grit Biotechnology Co., Ltd. The remaining authors declare that the research was conducted in the absence of any commercial or financial relationships that could be construed as a potential conflict of interest.

## ETHICS STATEMENT

This study was approved by the Medical Ethics Committee of Obstetrics and Gynecology Hospital of Fudan University, with the Approval number 2021–07. Written informed consent was obtained from patients for use of tissue samples.

## Supporting information


Figures S1‐S5

Table S1

Table S2

## Data Availability

The datasets used and/or analyzed during the current study are available from the corresponding author on reasonable request.
