# Supplementary material for: Modulation of intracellular kinase signaling to improve TIL stemness and function for adoptive cell therapy
Source: Cancer Med. 2022 Aug 26;12(3):3313–27. doi: 10.1002/cam4.5095 (PMC9939193; doi:10.1002/cam4.5095)

Supplemental Figure 1

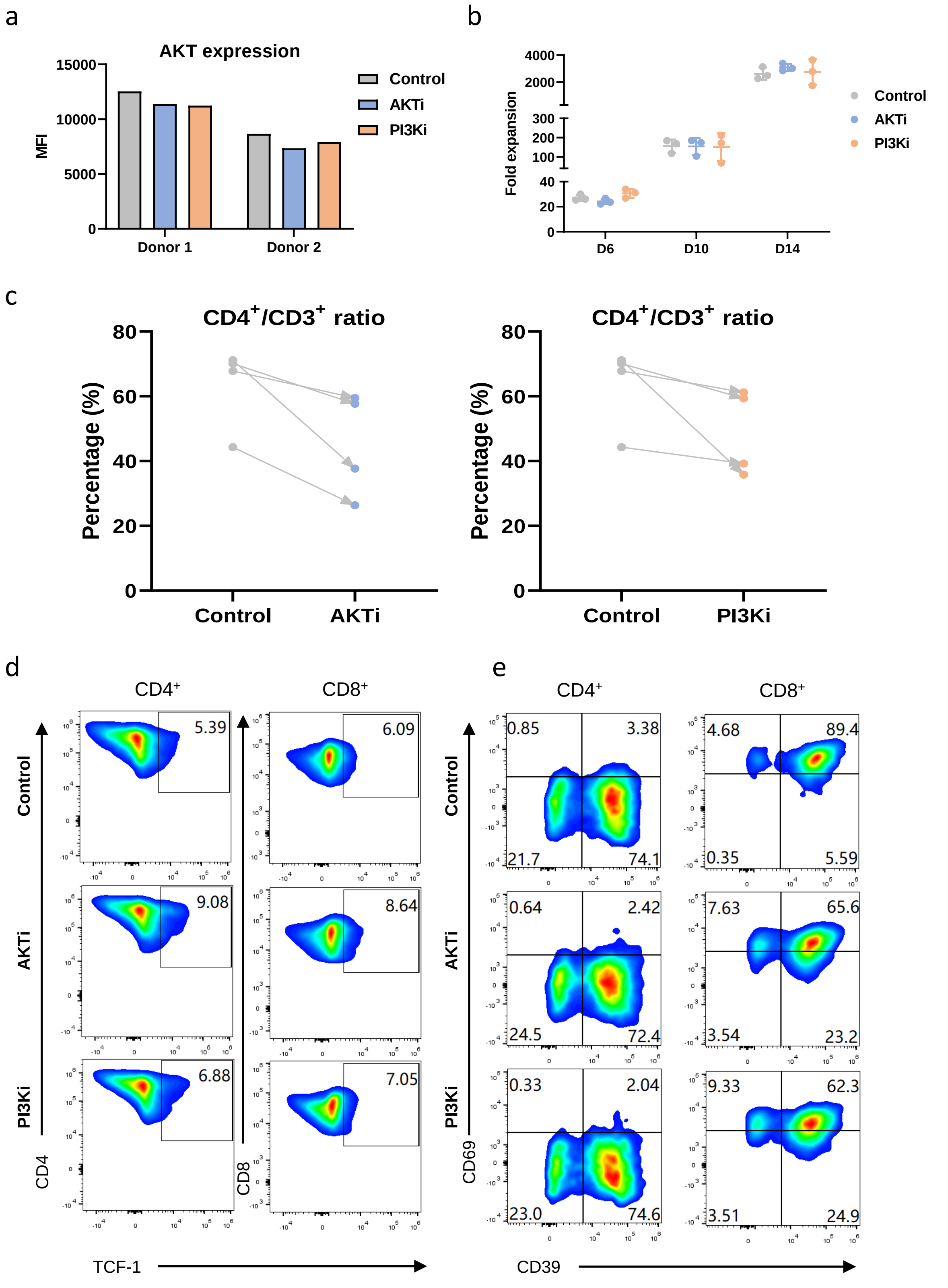

Supplemental Figure 2

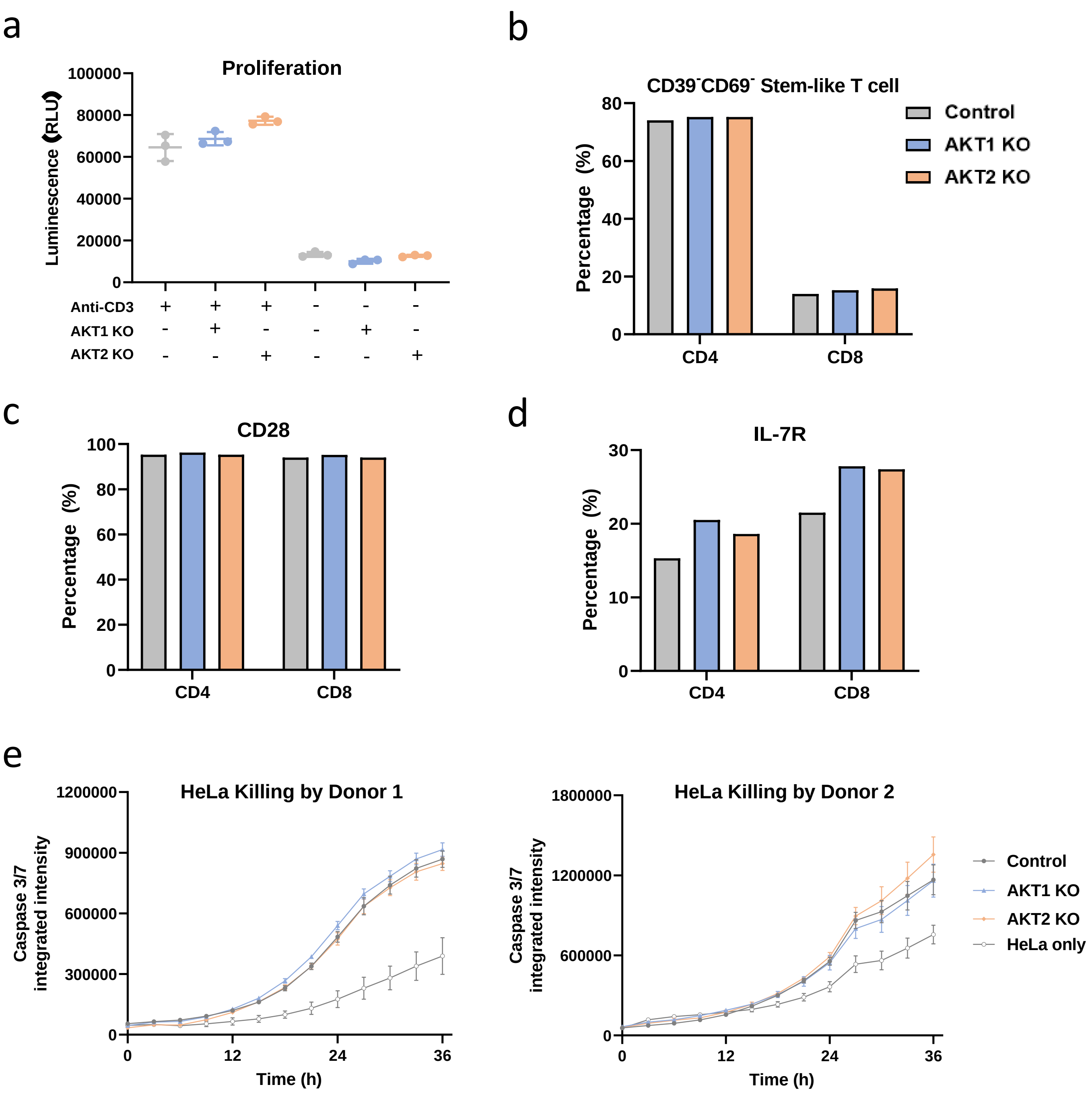

Supplemental Figure 3

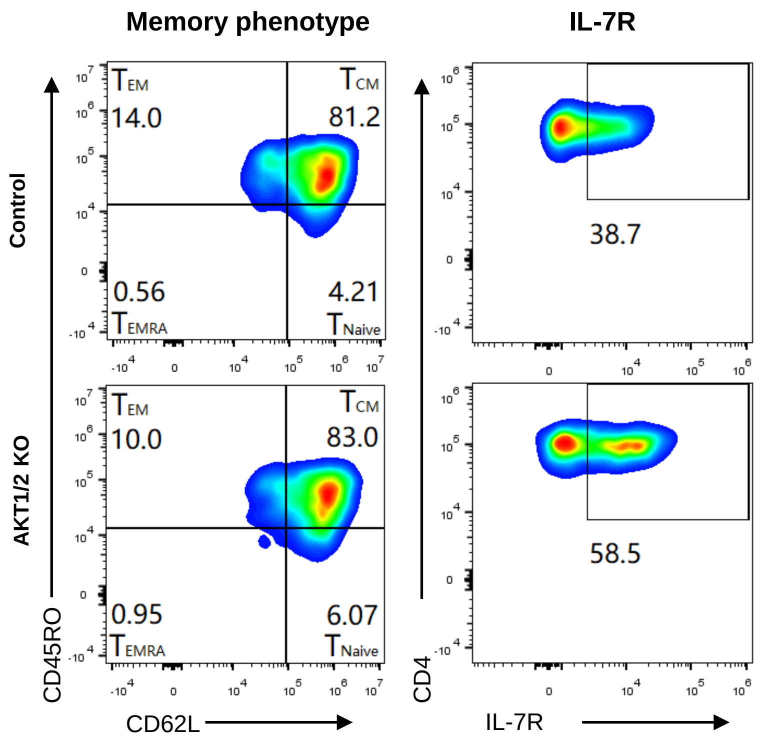

Supplemental Figure 4

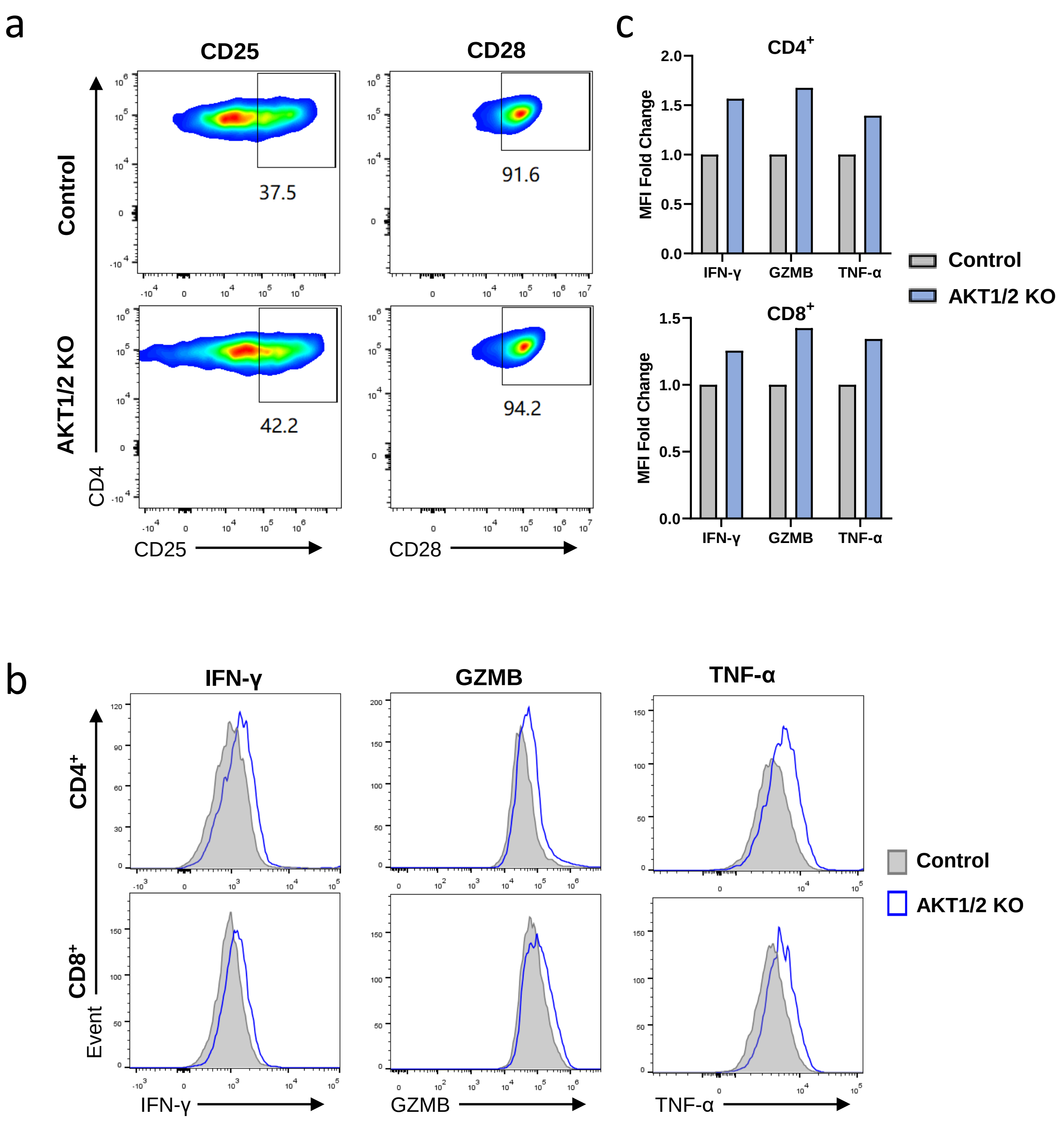

Supplemental Figure 5

a

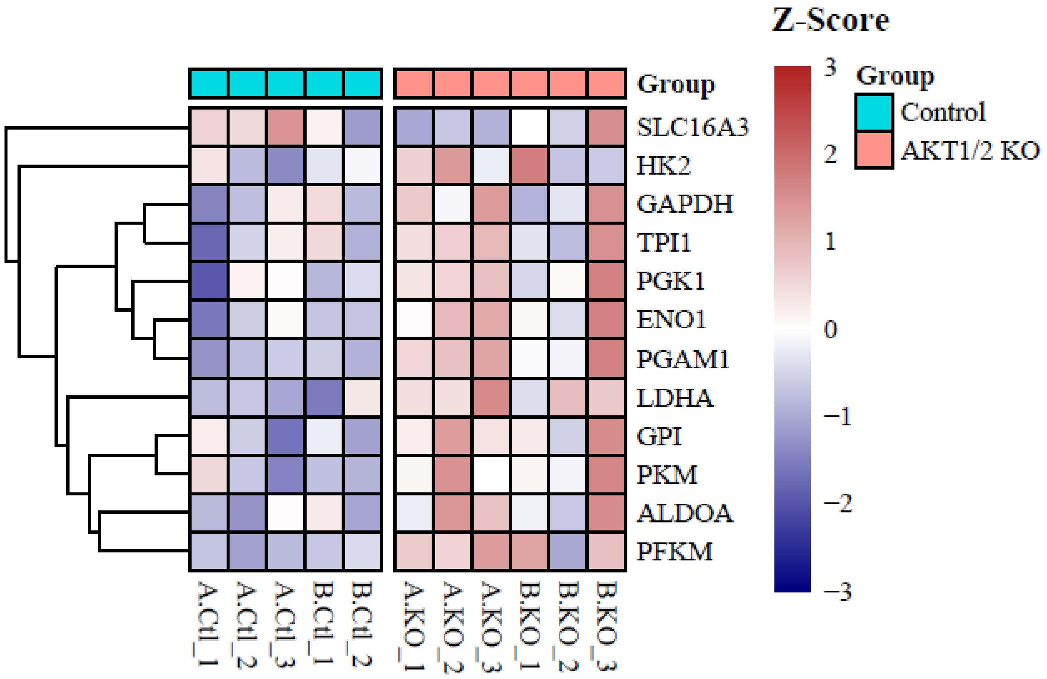

b

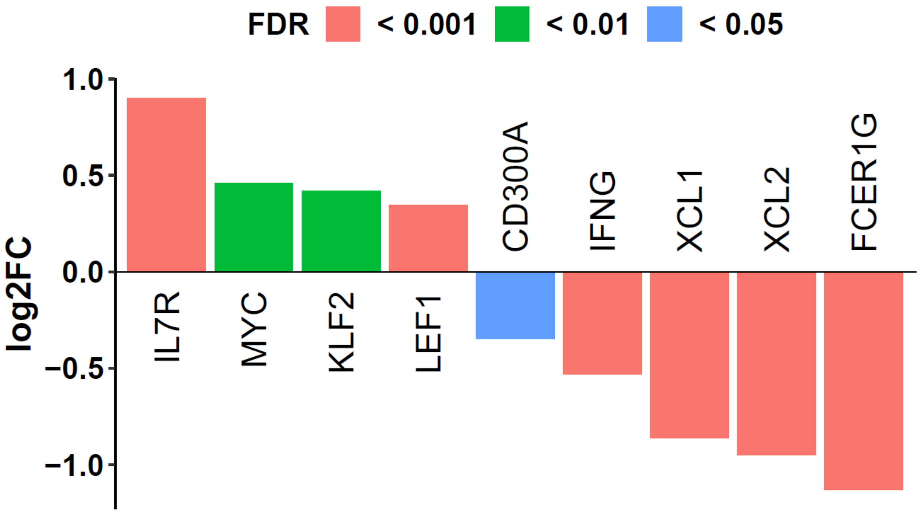

Supplement: Supplementary file 1 — Figures S1‐S5 [file CAM4-12-3313-s001.pdf]
